# Prognostic role of CD133 expression in colorectal cancer: a meta-analysis

**DOI:** 10.1186/1471-2407-12-573

**Published:** 2012-12-05

**Authors:** Ke Wang, Jianjun Xu, Junshu Zhang, Jian Huang

**Affiliations:** 1 Cancer Institute (Key Laboratory of Cancer Prevention & Intervention, National Ministry of Education; Provincial Key Laboratory of Molecular Biology in Medical Sciences), Second Affiliated Hospital, Zhejiang University School of Medicine, Hangzhou, 310009, China; 2 School of Finance, Zhejiang University of Finance and Economics, Hangzhou, 310009, China; 3 Department of Surgery, Linan People’s Hospital, Hangzhou, 311300, China

**Keywords:** CD133, Cancer stem cell, Colorectal cancer, Prognosis

## Abstract

**Background:**

CD133 has been identified as a putative cancer stem cell marker in colorectal cancer (CRC). However, the clinical and prognostic significance of CD133 in CRC remains controversial.

**Methods:**

Publications were identified which assessed the clinical or prognostic significance of CD133 in CRC up to October 2012. A meta-analysis was performed to clarify the association between CD133 expression and clinical outcomes.

**Results:**

A total of 12 studies met the inclusion criteria, and comprised 3652 cases. Analysis of these data showed that CD133 was not significantly associated with the depth of CRC invasion (odds ratio [OR] = 1.44, 95% confidence interval [CI]: 0.77–2.68, Z = 1.15, *P* = 0.252) or tumor differentiation (OR = 0.63, 95% CI: 0.28–1.46, Z = −1.06, *P* = 0.286). Also, there was no statistically significant association of CD133 with lymph node metastasis (OR = 1.16, 95% CI: 0.87–1.54, Z = 1.05, *P* = 0.315) or lymphatic invasion (OR = 1.08, 95% CI: 0.81–1.43, Z = 0.53, *P* = 0.594). However, in identified studies, overexpression of CD133 was highly correlated with reduced overall survival (relative risk [RR] = 2.14, 95% CI: 1.45–3.17, Z = 3.81, *P* = 0.0001).

**Conclusions:**

CD133 may play an important role in the progression of CRC, and overexpression of CD133 is closely related with poorer patient survival. If these findings are confirmed by well-designed prospective studies, CD133 may be a useful maker for clinical applications.

## Background

Colorectal cancer (CRC) is one of the most common visceral malignancies and a leading cause of death worldwide. Increasing evidence suggests that cancers, including CRC, may be hierarchically organized, with only a small population of cancer cells, termed cancer stem cells (CSCs), possessing the potential to initiate and sustain tumor growth and metastasis [1-3]. Furthermore, these cells are inert to toxic environmental agents owing to their high expression of ABC transporters, resistance to apoptosis, and efficient DNA repair mechanisms [4-6]. Thus, it is of major importance to investigate CSCs associated with cancer progression as they may be important factors in determining the clinical outcomes of cancer.

The cell surface marker CD133 (also known as prominin-1) is a five-domain transmembrane molecule, and has been identified as a putative CSC marker in various cancers, including brain tumors, prostate carcinoma and CRC [7-9]. Ricci-Vitiani *et al.* reported that CD133+ cells in CRC exhibited CSC properties *in vitro* and *in vivo*, such as self-renewal and high tumorigenic potential [10]. As few as 262 CD133+ CRC cells were able to form a tumor in NOD/SCID mice, whereas 10,000 CD133- cells failed. These suggested that CSCs had the ability to self-renew and to form the bulk of a tumor cell population.

The clinical relevance of CD133 is as a putative CSC marker in patients with CRC, where CSCs are thought to contribute to tumor progression and therapy resistance. Galizia *et al.* demonstrated that CD133 expression was correlated with clinical outcomes [11]. Overexpression of CD133 was significantly associated with malignant transformation or poor clinicopathologic parameters in CRC. However, Kojima *et al.* showed that CD133 expression varied according to the histological type of cancer [12]. There is insufficient clinical data to confirm a clinical application for CD133. In order to address controversial issues, we performed a meta-analysis to determine the association between CD133 expression and clinicopathologic parameters.

## Materials and methods

### Publication search

Publications were identified in the PubMed database (http://www.ncbi.nlm.nih. gov/pubmed/) using the following search terms: “CD133”, “colon cancer” or “colorectal cancer”, and “overall survival” or “OS”. Additional relevant searches were identified by manually cross-referencing abstracts of articles. Articles in this study were published up to October 2012. Titles and abstracts were evaluated to identify relevant publications, and the full text version scanned. The criteria for inclusion were: (1) articles dealing with CD133 expression and either prognostic factors or overall survival (OS) of CRC; (2) articles containing sufficient data to allow the estimation of an odds ratio (OR) or a relative risk (RR) of OS; (3) articles in the English language; and (4) articles published as original research. Reviews, comments, duplicated studies, and articles unrelated to our analysis were excluded. Studies with fewer than 50 patients, follow-up less than 2 years, and relevant articles using RT-PCR were also excluded.

The following information was extracted from the included papers: author, publication year, patient's country, tumor stage, number of patients, research technique used, antibody used, cutoff value of CD133, and tumor site. Two major groups were created according to the objective. One clarified the association between CD133 expression and clinicopathological parameters, including depth of invasion, degree of differentiation, lymph node status and lymphatic invasion. Another group investigated the association between CD133 expression and OS.

### Statistical analysis

The meta-analysis was performed as previously described [13]. For ease of analysis, the following data of CD133 expression and clinicopathological factors were combined into single categories: CD133-negative and low; T_1_ and T_2_ stages; T_3_ and T_4_ stages; and well and moderate differentiation. ORs with 95% CI were used to evaluate the association between CD133 expression and clinicopathological factors, including depth of invasion, differentiation, lymph node status and lymphatic invasion. Survival data were extracted from original papers as described by Parmar *et al.*[14]. Differences between CD133 expression and OS were quantified using RR with 95% CI. Heterogeneity across studies was evaluated with the Q test and *P* values. ORs and RRs were calculated by a random-effects model when the *P* value was less than 0.05. Otherwise, a fixed-effects model was used. Sensitivity analyses were performed to estimate the influence of individual studies on the summary effect. Funnel plots and Egger’s regression test was used to assess publication bias. Statistical analyses were estimated using R/meta software. *P* values were two-sided, with significance at *P* < 0.05.

## Results

### Description of studies

A total of 12 publications met the criteria for this analysis (Additional files 1 and 2) [12,15-25]. The total number of patients was 3652, ranging from 73 to 1235 patients per study. Main characteristics of the eligible studies were summarized in Table 1. Nine articles dealt with clinicopathological factors. Nine studies determined with OS. Three studies only reported the association between CD133 expression and clinicopathological factors without OS analysis. There were mainly two kinds of methods used to evaluate CD133 expression in CRC specimens: immunohistochemistry (IHC) and tissue microarray.

**Table 1:** Main characteristics of the eligible studies

**Study**	**Patient's country**	**Year**	**TNM grading/UICC staging**	**Technique**	**Number of patients**	**Antibody used, dilution**	**Cutoff of CD133 positive**	**Site**
Coco	Italy	2012	I–III	IHC	137	Santa Cruz 100	> 5%	Colon or Rectum
Hongo	Japan	2012	I–IV	IHC	303	Miltenyi Biotec 100	> 5%	Colon or Rectum
Zhang	China	2012	II–III	IHC	125	Novus 150	≥Score 4	colon
Xi	China	2011	I–IV	IHC	201	Abcam 200	≥Score 5	Colon or Rectum
Lugli	Switzerland	2010	I–IV	Tissue microarray	1235	Cell Signaling 100	> 5%	Colon or Rectum
Takahashi	Japan	2010	I–IV	IHC	151	Abcam 200	> 50%	Colon or Rectum
Ong	Australia	2010	I–IV	Tissue microarray	501	Miltenyi Biotech 10	> 5%	Colon or Rectum
Li	China	2009	IIIB	IHC	104	Abcam 150	≥5%	Colon or Rectum
Horst	Germany	2009	I–II	IHC	110	Cell Signaling 100	≥50%	Colon
Choi	South Korea	2009	0–IV	Tissue microarray	523	Santa Cruz 50	--	Colon or Rectum
Wang	China	2009	0–IV	IHC	73	Abcam 200	> 10%	Rectal
Kojima	Japan	2008	I–IV	IHC	189	Miltenyi Biotech 100	> 10%	Colon or Rectum

### Correlation of CD133 expression with clinicopathological parameters

**Figure 1:**
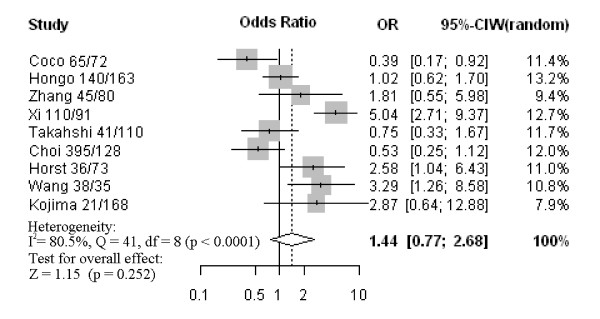
Forrest plot of odds ratios (ORs) for the association of CD133 expression with depth of invasion.

**Figure 2:**
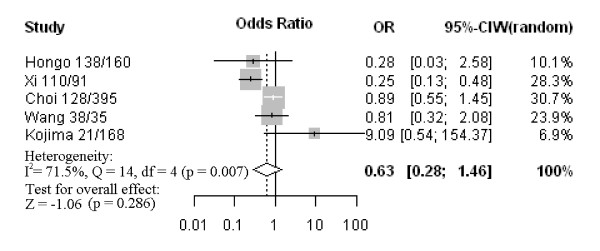
Forrest plot of ORs for the association of CD133 expression with tumor differentiation.

**Figure 3:**
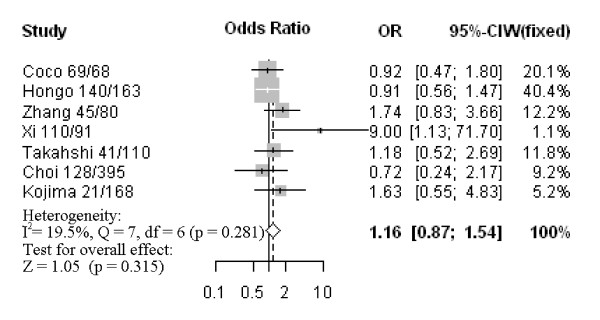
Forrest plot of ORs for the association of CD133 expression with lymph node metastasis.

**Figure 4:**
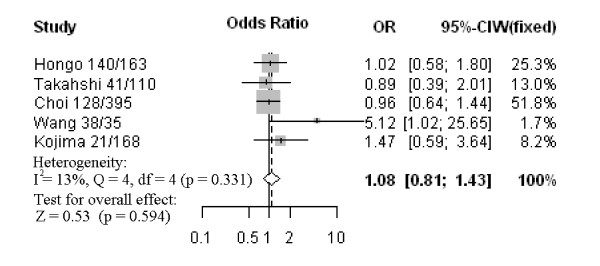
Forrest plot of ORs for the association of CD133 expression with lymphatic invasion.

Nine studies assessed the relationship between CD133 phenotype and depth of invasion (Figure 1). The pooled OR was 1.44 (95% CI: 0.77–2.68, Z = 1.15, *P* = 0.252 random-effect), and there was no significant heterogeneity, suggesting CD133 expression was not associated with depth of invasion. Five studies investigated the relationship between CD133 expression and degree of differentiation (Figure 2). There was no statistically significant association of CD133 and tumor differentiation (pooled OR = 0.63, 95% CI: 0.28–1.46, Z = −1.06, *P* = 0.286 random-effect). There was also no association between CD133 expression and clinical parameters such as lymph node metastasis (pooled OR = 1.16, 95% CI: 0.87–1.54, Z = 1.05, *P* = 0.315 fixed-effect) (Figure 3) or lymphatic invasion (pooled OR = 1.08, 95% CI: 0.81–1.43, Z = 0.53, *P* = 0.594 fixed-effect) (Figure 4). Moreover, Egger’s test indicated that above clinicopathological parameters showed no significant publication bias (Additional file 3).

### Impact of CD133 expression on overall survival of colorectal cancer

**Figure 5:**
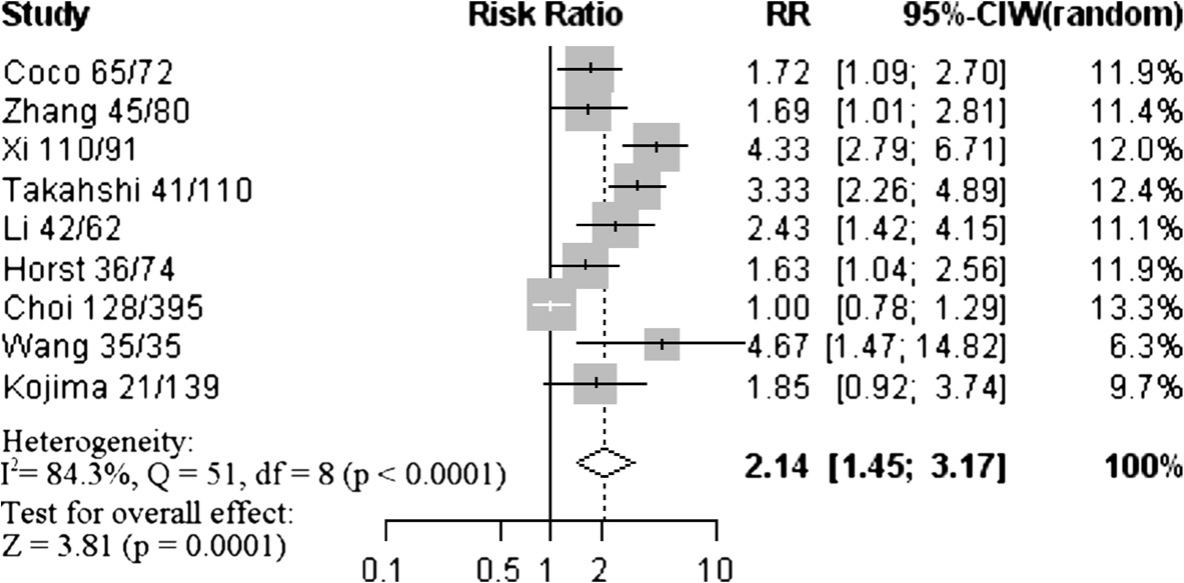
**Forrest plot of relative risk (RR) for the association of CD133 expression with overall survival (OS).** It showed that CD133 could be an independent prognostic factor for OS in CRC patients.

The meta-analysis was performed on nine studies (1581 patients) investigating the association of CD133 expression and OS. The pooled RR was calculated using the methods described above. As the test for heterogeneity was significant (*P* < 0.0001), a random-effect model was used to calculate the RR. The presence of CD133 expression was highly correlated with poor OS (pooled RR = 2.14, 95% CI: 1.45–3.17, Z = 3.81, *P* = 0.0001) (Figure 5). This indicated that CD133 was an independent prognostic factor in CRC. No significant publication bias was detected (Additional file 3), and the explanatory variables did not significantly influence RR estimates for OS (Additional file 4).

## Discussion and conclusion

To our knowledge, the present meta-analysis is the first English language study to systematically determine the association between CD133 expression and CRC survival. As the prognostic and predictive significance of the CD133 phenotype in CRC was controversial, a quantitative meta-analysis of the study outcomes was warranted. Our analysis indicated that CD133 expression was significantly associated with OS, indicating that it might be a marker for poor prognosis of CRC.

CD133 is the epitope of a glycosylated form of membrane protein, and the physiological function of CD133 remains unknown. It was originally classified as a potential CSC marker in CRC. Ieta *et al.* reported that CRC tissue had much higher CD133 protein expression than that of normal tissue [26]. In our study, the data showed that CD133 was not positively correlated to the depth of invasion or degree of tumor differentiation. Although lymph node metastasis and lymphatic invasion were more common in cases with high CD133 than in those with CD133-negative or low, the differences were not statistically significant. Concerning the prognostic value of CD133, a few papers reported significant associations with CRC, and that CD133 might be considered as a new marker to determine the stage and management of CRC [20,27]. It is notable that this association is observed in our meta-analysis of CD133 phenotype and OS, suggesting that this marker can be developed for clinical applications.

For future studies, co-expression of colorectal CSC markers associated with patient survival may be more meaningful for clinical application in CRC. Several studies have shown that CSC-related factors, including ALDH1 and LGR5, are associated with cancer progression [28,29]. In addition, CSCs have major phenotypic and functional heterogeneity which may help distinguish them from cancer cells, and may be of potential benefit in the development of anti-cancer therapies to improve clinical outcomes [30].

Certain limitations exist in this meta-analysis. First, OS was determined from unadjusted RRs in the published papers, and RRs from the survival curves might be less reliable than direct analysis of variance. Ideally, measurements should be obtained directly from published statistics and adjusted using other prognostic factors. Second, the patient populations were not uniform, e.g., Choi *et al.* focused on patients with invasiveness and differentiation of CRC [25], while all the patients in the Ong *et al.* study received neoadjuvant chemotherapy [19]. These differences might partly influence the significance of the clinicopathological outcome in survival analyses.

In summary, this meta-analysis indicated that CD133 expression was not associated with common clinical parameters of CRC, such as depth of invasion, tumor differentiation, lymph node metastasis and lymphatic permeation. However, high CD133 expression was associated with a worse outcome than CD133-negative or -low expression, and CD133 was an independent factor associated with reduced survival. Further studies of CD133 and its potential as a marker for CRC prognosis in clinic are warranted.

## Abbreviations

CI: Confidence interval; CRC: Colorectal cancer; CSC: Cancer stem cell; IHC: Immunohistochemistry; OR: Odds ratio; OS: Overall survival; RR: Relative risk.

## Competing interests

The authors declare that they have no competing interests.

## Authors' contributions

KW participated in extracting the data and wrote the manuscript. JX performed the statistical analysis. JH and JZ carried out literature search and data collection. All authors approved the final manuscript.

## Pre-publication history

The pre-publication history for this paper can be accessed here:

http://www.biomedcentral.com/1471-2407/12/573/prepub

## Supplementary Material

Additional file 1 PRISMA 2009 Flow Diagram. Click here for file

Additional file 2 PRISMA 2009 Checklist. Click here for file

Additional file 3 Egger's test of funnel plot asymmetry. Click here for file

Additional file 4 Results of meta-regression analysis exploring source of heterogeneity with overall survival. Click here for file
