# Supplementary material for: Prognostic role of CD133 expression in colorectal cancer: a meta-analysis
Source: BMC Cancer. 2012 Dec 5;12:573. doi: 10.1186/1471-2407-12-573 (PMC3532409; doi:10.1186/1471-2407-12-573)
Supplement: Additional file 1 — PRISMA 2009 Flow Diagram. [file 1471-2407-12-573-S1.doc]

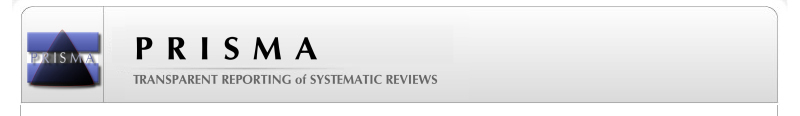
**PRISMA 2009 Flow Diagram**

r

**Screening**

**Included**

**Eligibility**

**Identification**

Records identified through database searching
(n =67)

Additional records identified through other sources
(n = 4)

Total research results
(n = 71)

Relevant citations
(n = 32)

Duplicated citations
(n = 3)

Relevant citations
(n = 29)

Excluded on title/abstract review citations: review/editorial/ letter/commentary (n = 5)

Candidate studies for full-text review:
(n = 24)

Studies included in the meta-analysis:
(n = 12 )

Irrelevant citations
(n = 39)

Excluded on full-text review:
(n = 12)
