# Supplementary material for: Prognostic role of CD133 expression in colorectal cancer: a meta-analysis
Source: BMC Cancer. 2012 Dec 5;12:573. doi: 10.1186/1471-2407-12-573 (PMC3532409; doi:10.1186/1471-2407-12-573)
Supplement: Additional file 3 — Egger's test of funnel plot asymmetry. [file 1471-2407-12-573-S3.docx]

Table S1 Egger's test of funnel plot asymmetry.

| Clinicopathological parameters | t value | df | *P* value |
| --- | --- | --- | --- |
| Depth of invasion | 0.25 | 7 | 0.81 |
| Degree of differentiation | 0.25 | 3 | 0.82 |
| Lymph node metastasis | 2.00 | 5 | 0.10 |
| Lymphatic invasion | 2.38 | 3 | 0.10 |
| Overall survival | 1.82 | 7 | 0.11 |
