# Supplementary material for: Prognostic role of CD133 expression in colorectal cancer: a meta-analysis
Source: BMC Cancer. 2012 Dec 5;12:573. doi: 10.1186/1471-2407-12-573 (PMC3532409; doi:10.1186/1471-2407-12-573)
Supplement: Additional file 4 — Results of meta-regression analysis exploring source of heterogeneity with overall survival. [file 1471-2407-12-573-S4.docx]

Table S2 Results of meta-regression analysis exploring source of heterogeneity with overall survival.

| Covariates | Univariate analysis | | |
| --- | --- | --- | --- |
|  | Coefficient | SE | *P* value |
| Tumor stage | -0.06 | 0.07 | 0.40 |
| Antibody used | 0.04 | 0.06 | 0.50 |
| Cutoff of CD133 positive | 0.02 | 0.08 | 0.84 |

Note: The dependent variable is the RR for overall survival (OS) from each study. Weights have been assigned according to the estimated variance of RR. SE, standard error of the coefficient.
